# Adolescent and Youth Sexual Reproductive Health (AYSRH): Perceived Religious Health Assets of Churches and Their Optimization for Youth Sexual Health in South Africa’s Vaal Region

**DOI:** 10.3390/healthcare14101289

**Published:** 2026-05-09

**Authors:** Vhumani Magezi

**Affiliations:** 1 URT Research Unit, Faculty of Theology, North-West University, Potchefstroom 2531, South Africa; vhumani.magezi@nwu.ac.za; 2 Oxford Institute of Population and Ageing, University of Oxford, Oxford OX2 6PR, UK

**Keywords:** adolescent and youth sexual and reproductive health, AYSRH, church health assets, religious health assets, faith-based organisations, South Africa

## Abstract

**Background:** The role of religion and faith-based organisations in public health is increasingly examined through the framework of religious health assets (RHAs), defined as resources located in or held by religious entities that may be mobilised for health and development. Within this framework, church health assets (CHAs) are conceptualised as congregationally specific expressions of RHAs, namely, the tangible and intangible resources recognised within local church settings and interpreted by church leaders as relevant to adolescent and youth sexual and reproductive health (AYSRH). Despite growing interest, there remains limited empirical work examining how such assets are perceived in relation to young people’s sexual and reproductive health, particularly from an emic perspective in sub-Saharan Africa. **Aim:** This study explored how pastors in South Africa’s Vaal Triangle perceive church assets relevant to AYSRH. **Methods:** The article presents findings from a qualitative study based on in-depth semi-structured interviews with eleven purposively selected pastors from Vanderbijlpark, Vereeniging, and Sasolburg. Data were collected between August 2019 and February 2020, prior to the COVID-19 restrictions that later altered face-to-face engagement in South Africa. Data were analysed using thematic content analysis informed by interpretive description, employing iterative coding, constant comparison, memoing, and a clearly defined audit trail. **Results:** The findings identified ten perceived CHAs, comprising five tangible assets, interaction spaces, community resources, normative teaching materials, networks and partnerships, and financial resources—and five intangible assets—reputation, voice on sexuality, mission and vision, a ready audience, and embodied messages. Across these themes, pastors predominantly framed AYSRH in moral and pedagogical terms, emphasising abstinence, guidance, and restoration, rather than a broader continuum encompassing information, prevention, care, rights, and service access. **Conclusions:** The study concludes that pastors perceive churches to possess substantial AYSRH-related assets; however, the analysis reflects perceptions rather than demonstrated implementation or measurable impact. The findings highlight both potential and limitation, indicating that the same assets may function as facilitators or barriers depending on their interpretation and application. The study contributes a pastor-centred, emic account of CHAs within a South African context and underscores the need for future multi-stakeholder research to assess how faith-sensitive AYSRH interventions operate in practice.

## 1. Introduction

The relationship between religion, public health, and development is increasingly understood through the language of religious health assets (RHAs)**,** a concept used to describe resources held within religious traditions, institutions, networks, and practices that may contribute to health and well-being [1,2,3,4]. At the same time, adolescent and youth sexual and reproductive health (AYSRH) remains a pressing concern in many African settings, including South Africa, where young people continue to navigate high levels of HIV vulnerability, unintended pregnancy, uneven access to sexuality education, and conflicting moral and institutional messages about sexuality [5,6,7,8].

Churches are especially important to this discussion because they remain dense social institutions in many African communities and often command trust, moral authority, physical infrastructure, and volunteer networks [2,9,10]. Yet their role in AYSRH is not straightforward. Faith communities may create supportive spaces for mentoring, belonging, and guidance, but they may also reproduce silence, stigma, exclusion, or narrow moral framings around sex and sexuality [7,11,12,13,14]. This ambivalence makes it necessary to study not only whether churches matter for youth health, but also how church actors themselves understand the assets they possess and the limits of those assets.

Conceptually, this article distinguishes between RHAs and CHAs. RHAs refer to the broader category of health-relevant resources located in or associated with religious entities, traditions, and actors [2,3]. CHAs, by contrast, are used here as a more specific analytic term for the tangible and intangible assets recognised within local church life and articulated by church leaders in relation to AYSRH. CHAs are therefore not a wholly separate concept from RHAs; rather, they are a congregationally focused and empirically delimited expression of the broader RHA framework.

The need for such clarification is important because the RHA literature, while influential, continues to face challenges of definitional ambiguity, uneven evidence, and limited evaluative work that can guide policy [3,4,15]. These challenges are amplified in the domain of AYSRH, where relatively few studies focus specifically on young people and where debates about sexuality often reveal deep tensions between public-health, rights-based, educational, and theological frameworks [4,7,11]. In South Africa, this tension is particularly salient. Research on youth, sexuality, and education has shown that young people encounter sexuality through complex intersections of gender, inequality, HIV, school policy, media, and religion rather than through a single institutional script [7,11,12,16].

The broader international literature also indicates that religious spaces beyond churches are being reworked as sites of sexuality education and contestation. For example, Ben-Lulu’s study of Reform Jewish settings in the United States shows how sexuality education in synagogue contexts can be reinterpreted through more inclusive theological language, including queer-affirming pedagogies [17]. This wider comparative literature is important because it demonstrates that the relationship between faith and sexuality education is not fixed; rather, it is historically and institutionally negotiated across religious traditions.

This article addresses a specific and narrower gap within that broader field. Rather than claiming to assess AYSRH outcomes or intervention effectiveness, it explores what pastors in one South African region perceive to be church health assets for AYSRH, and how those perceptions illuminate both opportunities and limits for faith–public health engagement. The study adopts an emic perspective because much of the literature on religion and health has been written from etic standpoints that privilege external development or policy framings over faith communities’ own understandings [1,3,15]. By focusing on pastors’ perceptions, this article contributes empirical detail to ongoing debates about what counts as a religious health asset, who defines it, and under what conditions such an asset may support or constrain youth sexual and reproductive health.

## 2. Literature Review

### 2.1. Faith-Based Organisations, Religion, and AYSRH

AYSRH refers to physical, mental, emotional, and social well-being in relation to sexuality and reproduction among adolescents and young adults. Internationally, this includes access to information, comprehensive sexuality education, prevention and treatment services, family planning, HIV and STI prevention, and supportive environments for informed decision-making [5,6]. Addressing AYSRH therefore requires more than biomedical provision; it requires engagement with the social institutions that shape norms, relationships, belonging, and moral authority.

Faith-based organisations are among those institutions. Their relevance is visible in global development policy, where religion has increasingly re-entered debates on partnership, inclusion, and service delivery [1,18]. In many communities, faith actors provide health facilities, community outreach, informal counselling, social support, and mobilisation capacity [9,19]. The influential Lancet series on faith-based healthcare similarly highlighted the practical importance of partnerships between the public sector and faith actors in low- and middle-income settings [9,20].

At the same time, the intersection of religion and sexuality is frequently marked by contestation. Landry et al. showed that faith- and community-based organisations may support sexuality education, yet their programmes are often shaped by tension between comprehensive sexual health goals and moral or doctrinal commitments [21]. In South Africa, debates about sexuality education are similarly embedded in broader struggles over gender norms, moral order, heteronormativity, and youth citizenship [7,11,12]. Bhana and Pattman argued that research on South African youth sexuality must take seriously the context of HIV, gendered power, and unequal social conditions rather than reducing young people’s experiences to individual behaviour alone [11]. Bhana, Crewe, and Aggleton likewise emphasised that sexuality and education in South Africa should be understood through layered histories of inequality, schooling, and competing normative regimes [7].

These insights are highly relevant for interpreting church-based AYSRH discourse. If sexuality is socially produced through institutions, media, peer cultures, and structural inequality, then church discourse is only one among several meaning-making systems encountered by young people. Francis’s work with queer South African youth further shows that young people may experience standard sexuality education as exclusionary when it fails to address sexual diversity, lived complexity, and the need for recognition [12]. Cloete similarly argued that faith communities have a potential role in sexuality education, but only if they engage youth culture critically and responsibly rather than simply repeating moral prohibitions [16].

### 2.2. Religious Health Assets and Church Health Assets

The African Religious Health Assets Programme (ARHAP) helped formalise the concept of RHAs by defining them as assets located in or held by religious entities that can be leveraged for development or public health [2]. This framing is consistent with wider health-assets scholarship, which emphasises resources that enhance the capacity of individuals and communities to maintain health and well-being [4,22]. Within this perspective, assets may be material, social, symbolic, organisational, or spiritual.

Churches can plausibly be understood as sites where such assets cluster. Their buildings may function as meeting places and shelters; their networks may enable mobilisation; their leaders may provide trusted communication; and their shared narratives may shape motivation, belonging, and care [10,19]. This is one reason churches have been important in many African responses to HIV and community health [10,23].

However, the literature also cautions against treating religious assets as self-evidently beneficial. Scholars have noted that the term asset may become overly celebratory or instrumental if it obscures ambivalence, conflict, or exclusion [3,4]. What counts as an asset depends on perspective, context, and use. An institution’s moral authority may facilitate uptake of health messages in one context while intensifying stigma in another. A close-knit faith community may offer support to some youth while marginalising others, particularly those whose sexual practices or identities do not align with dominant norms [12,17,24].

For this reason, the value of the CHA concept in the present study is not that it proves churches are effective AYSRH providers. Rather, it enables a more precise empirical question: which resources do pastors themselves identify as relevant to AYSRH, and how are those resources morally and institutionally framed? That narrower focus helps address a key gap in the RHA literature, namely the need to understand what is perceived as an asset, by whom, and with what practical implications [4].

### 2.3. Study Focus and Contribution

Several gaps motivate this study. First, there is limited empirical work on RHAs in relation to young people’s health, and especially on AYSRH in sub-Saharan Africa [4]. Second, the literature continues to call for more self-contained and methodologically transparent qualitative studies capable of clarifying how assets are identified and interpreted in practice [3,4,15]. Third, South African scholarship shows that sexuality education and youth sexual citizenship are deeply contested, yet relatively little work has examined how pastors specifically interpret church capacities within that contested terrain [7,11,12,16].

This article contributes by providing an emic, pastor-centred account of perceived CHAs in the Vaal Triangle. Its contribution is therefore analytical rather than evaluative: it does not test implementation or measure AYSRH outcomes, but clarifies how church leaders construct the idea of health-relevant assets and what those constructions imply for collaboration with public-health actors.

## 3. Methodology

### 3.1. Study Design and Paradigmatic Orientation

This article forms part of a larger qualitative study on church-led AYSRH interventions in South Africa’s Vaal Triangle. The present paper reports a stand-alone analysis of the pastor interview data only. The wider project was informed by a pragmatic paradigm and used interpretive description as its qualitative design. Pragmatism was appropriate because the study was concerned with a practical problem—how churches understand resources relevant to AYSRH in a context of HIV risk, pregnancy, and contested sexuality norms—and with the consequences of those understandings for real-world engagement. Interpretive description was chosen because it is designed for applied fields where the aim is not abstract theory building alone but analytically grounded insight for practice-oriented questions [25,26].

### 3.2. Setting and Sampling Strategy

The study was conducted in the Vaal Triangle, which includes Vanderbijlpark, Vereeniging, and Sasolburg. This region was selected because it combines a substantial church presence with longstanding concern about HIV and youth sexual health.

Sampling was purposive. Churches were selected to capture variation in theology, denominational orientation, racial composition, and local context. Inclusion criteria required churches to have at least 100 members, a substantial youth presence, a significant number of parents attending with children or youth under their care, and a senior pastor willing to facilitate the broader study. From an initial pool of 21 churches meeting these criteria, 11 were selected through researcher judgement to ensure variation across racial composition and church orientation. Two churches were classified as relatively open or accommodative on AYSRH matters, four as conservative, and five as Pentecostal. Seven pastors led predominantly Black African congregations, while four led mixed congregations that were predominantly White. The sample included at least one female pastor, although detailed demographic data such as age and years of pastoral service were not systematically collected for this paper. Table 1 summarises the interviewed pastors.

The sample size of eleven pastors was considered adequate for this qualitative aim because the study sought depth and comparative variation rather than statistical representation. Sampling was guided by information power: the participants held institutionally central roles, the study question was focused, and the later interviews did not generate substantively new asset categories beyond those already emerging in earlier interviews. In this sense, analytic sufficiency was reached when the core tangible and intangible asset patterns became repetitive across church traditions and sites.

### 3.3. Data Collection

Data were collected between August 2019 and February 2020. Importantly, the interviews were completed before the COVID-19 lockdown and the major distancing restrictions introduced in South Africa in late March 2020. The pandemic therefore did not directly constrain the face-to-face interview interactions reported in this paper, although it later formed part of the broader context in which AYSRH service conversations continued nationally.

The first author conducted all eleven in-depth semi-structured interviews. The interview guide was aligned to the study objectives and included probing questions on church resources, perceived youth sexual health needs, the role of pastors and parents, church teaching on sexuality, and possibilities for broader intervention. Interviews lasted approximately 60–80 min, were audio-recorded with consent, and were supplemented with field notes.

### 3.4. Data Analysis

The pastor interviews were analysed through thematic content analysis informed by interpretive description [25,27]. The first author conducted the coding. Analysis followed an iterative six-stage process adapted from Braun and Clarke: familiarisation with the transcripts; generation of initial codes; grouping of codes into candidate themes; review and refinement of themes; definition and naming of themes; and production of the final analytic narrative [27].

Coding was not treated as a rigid mechanical exercise. Instead, transcripts were repeatedly read and re-read to identify similarities, differences, emphases, silences, and contradictions across participants. Constant comparison was used within and across interviews to test the consistency of developing interpretations. Memos were kept to document analytic decisions, and pseudonyms (P1–P11) were used throughout to preserve confidentiality while maintaining a clear analytic trail from quotation to interpretation.

No second independent coder was used for this analysis. To strengthen rigor despite single-researcher coding, the analysis relied on repeated recoding, comparison across cases, explicit linking of claims to verbatim data, and a transparent account of the logic through which themes were generated.

### 3.5. Researcher Reflexivity and Rigor

Ethical approval was granted by the North-West University Basic and Social Sciences Research Ethics Committee (BaSSREC; approval number NWU-00879-19-S7). Additional gatekeeper permission was obtained through the Greater Vaal Pastoral Forum.

Reflexivity was particularly important in this study. The author is a trained and practising Practical Theologian with substantial experience in health development, HIV, and sexual and reproductive health programming. This proximity to the field was advantageous because it supported contextual sensitivity, familiarity with church discourse, and the ability to recognise nuanced distinctions among congregational positions. At the same time, such proximity also created a risk of over-reading the data through prior commitments or experience. The analysis therefore deliberately sought to “stand back” from premature conclusions by relying on thick description, generous quotation, constant comparison, and explicit memoing of interpretive decisions.

Rigor was strengthened through four linked strategies: epistemological coherence between paradigm, design, methods, and analysis; representative credibility through purposive variation and prolonged engagement across seven months of fieldwork; analytic logic through a documented coding trail and theme development process; and interpretive authority through close alignment between claims and the presented data. Because this paper analyses only one subset of the wider project, triangulation is limited within the article itself; accordingly, claims are confined to pastors’ perspectives.

## 4. Findings

The analysis identified ten perceived church health assets relevant to AYSRH. To improve clarity, the findings are organised into tangible and intangible assets and interpreted analytically rather than through quotation alone. Table 2 summarises the themes.

### 4.1. Tangible Assets

#### 4.1.1. Church Interaction Spaces

Pastors described church buildings as potentially safe and familiar spaces where young people could meet, learn, seek support, or be temporarily sheltered in situations of pregnancy, abuse, or vulnerability. They also referred to digital platforms such as WhatsApp and Facebook, along with mentorship structures, drama, and sport.

*“We have personal conversations with the youth as a form of mentorship and accountability. This encourages healthy conversations on sexual issues. The leaders make themselves available to the youth to support and encourage them.” (P6)*

Analytically, this theme shows that pastors imagined the church first as an accessible platform for contact rather than as a specialist AYSRH service provider. The emphasis was on availability, familiarity, and relational access. However, much of this language was aspirational, what churches can do, rather than evidence of routinised intervention infrastructure. This distinction is important because it marks a gap between perceived capacity and demonstrated implementation.

#### 4.1.2. Churches as a Community Resource

Participants identified parents, health professionals, committed congregants, and family networks as part of the church’s human-resource base. Such people were seen as important for teaching, counselling, referral, and buffering youth against risk.

*“Christian families are an asset… Parents should be taught about sexual reproductive health… Parents should develop relationships with young people, so that they open up on sexual reproductive health issues…” (P7)*

This theme suggests that pastors understood church assets relationally, not only institutionally. The church was imagined as a social ecology linking parents, mentors, and professionals. At the same time, the prominence of family language also reflects a normative assumption that youth support is best channelled through conventional family structures, an assumption that may not fully account for fractured households, queer youth experience, or young people who do not feel safe speaking within family settings [12].

#### 4.1.3. Normative Teaching Materials

The Bible was consistently described as the church’s central asset for dealing with sexuality. Pastors also referred to Bible studies and manuals that frame sexual behaviour through scriptural teaching, life skills, and abstinence-oriented guidance.

*“The Bible, as taught by churches, has an answer… So it is one of our greatest assets in teaching, rebuking and correcting.” (P11)*

This was one of the most important themes in the dataset because it clarifies that many pastors did not primarily conceptualise AYSRH as a field of services or rights, but as a domain of moral formation. The power of this asset lay not only in its content but in its authority. Yet this same strength may narrow the scope of engagement where sexuality is reduced to right and wrong conduct rather than addressed as a broader set of relational, informational, preventive, and care needs.

#### 4.1.4. Networks and Partnerships

Pastors described links to NGOs, social workers, and local organisations that could provide training or intervention support beyond the church’s own capacity.

*“Adolescent and youth sexual reproductive health is a responsibility for all people… We should use all the resources that are available, including partnering with different relevant stakeholders…” (P4)*

This theme indicates that pastors were not uniformly isolationist. There was clear recognition that church resources were insufficient on their own, especially for complex cases. Even so, the language of partnership remained largely instrumental and referral-oriented. The interviews offered less evidence of fully integrated partnerships in which church and public-health actors jointly redesign programme content or negotiate conflicting epistemologies of sexuality.

#### 4.1.5. Financial Resources

Some participants identified financial contributions, offerings, and better-resourced congregations as possible means of supporting workshops, camps, or expert-led programmes.

Financial resources were therefore perceived as enabling assets, but they were discussed unevenly and usually in relation to events rather than sustained programming. This suggests that financial capacity was recognised, but not framed as the primary driver of church engagement. Moral legitimacy, messaging authority, and audience access appeared more central than budgetary strength.

### 4.2. Intangible Assets

#### 4.2.1. Reputation

Pastors considered church reputation an important asset because messages communicated in church were perceived as truthful, authoritative, and trustworthy.

*“Something that is communicated in church is considered true and correct… The integrity of the church is one of our huge and greatest assets.” (P11)*

Reputation operated here as symbolic capital. It could support disclosure, trust, and uptake of messages. Yet the data also show that this authority was sometimes defined against secular health messaging, especially contraception discourse. Accordingly, reputation may strengthen communication while simultaneously narrowing the range of acceptable knowledge.

#### 4.2.2. Voice of the Church on Sexuality

Participants emphasised that the church has a distinctive voice on sexuality, often described as the “conscience” of society. This voice included counselling, mentoring, teaching, and attempts to provide therapeutic or restorative support.

*“The message of the church should be the conscience of the society on sexual issues, i.e., not having sex until you are married.” (P5)*

The pastoral voice was therefore double-sided. It included care, accompaniment, and opportunities for conversation, but it also functioned as a regulatory voice that sought to discipline sexual conduct. This dual quality helps explain why the same pastors could describe churches as supportive and yet still frame sexuality within a restrictive moral script.

#### 4.2.3. Mission and Vision

Many pastors understood engagement with AYSRH as part of the church’s God-given mission. Their sense of obligation centred on teaching the “right” view of sex, frequently expressed as abstinence before marriage.

Mission and vision gave AYSRH work theological legitimacy, but also fixed the parameters of what counted as faithful engagement. In practice, this meant that a commitment to young people’s well-being was often articulated through moral instruction rather than through broader service-oriented or rights-based AYSRH language.

#### 4.2.4. Church Audience

Participants saw congregational gatherings as providing a ready-made audience for repeated messaging and youth engagement. They also noted that the boundary between church members and the wider community was not always rigid.

This asset is analytically significant because it shows why churches remain attractive partners in public health: they offer routine access to audiences that many services struggle to reach consistently. Yet reach alone does not determine relevance. The effectiveness of that reach depends on the content, tone, inclusivity, and responsiveness of the messages being delivered.

#### 4.2.5. Embodied Messages

Pastors described testimonies, visible moral conduct, and stories of repentance or restoration as powerful forms of communication.

*“The Church is a living organism… The way we live our lives… is a message to the people.” (P2)*

This theme shows that for pastors, communication about AYSRH was not confined to formal teaching. It was also mediated through example, witness, and communal identity. Such embodied messages may be compelling because they personalise moral teaching. At the same time, they can privilege narratives of failure-and-redemption that make some experiences legible while rendering others, especially queer, dissenting, or non-repentant lives, less speakable within church spaces [12,17].

## 5. Discussion

This study set out to explore what pastors in the Vaal Triangle perceive to be church health assets relevant to AYSRH. The findings indicate that pastors identify a substantial range of tangible and intangible resources, many of which align with broader RHA literature: buildings, networks, social relationships, moral authority, trusted communication, and shared mission [2,3,4,9]. In that sense, the study supports the argument that churches cannot be understood merely as doctrinal institutions; they are also organisational, relational, and symbolic infrastructures with potential public-health relevance.

At the same time, the study also shows why the language of “assets” must be used carefully. The pastors’ accounts were not neutral inventories of resources. They were moral interpretations of what counts as a useful resource in the first place. In this dataset, an asset was often valuable because it reinforced biblical authority, supported abstinence, or preserved the church’s witness. This means that the same resource that public health actors may regard as a delivery channel can, from an emic church perspective, be valued primarily as a means of moral formation.

This distinction helps explain a major pattern in the findings: while pastors described multiple assets, they often narrowed AYSRH itself to a comparatively limited field of meaning. Rather than framing AYSRH broadly in terms of prevention, treatment, rights, access, and diversity, many pastors framed it as guidance on appropriate sexual conduct, protection from moral error, and pastoral restoration after sexual harm or transgression. Accordingly, the main contribution of the article is not to show that churches are ineffective, nor to romanticise them as naturally effective, but to demonstrate that the operational meaning of church assets is shaped by how sexuality is morally constructed.

This finding resonates with South African scholarship showing that sexuality education is not simply a technical matter of information transfer. It is bound up with gendered norms, institutional power, and struggles over who gets to define acceptable sexuality [7,11,12]. It also resonates with comparative faith-based literature showing that religious settings can interpret sexuality education in very different ways, ranging from restrictive moralism to more inclusive and dialogical approaches [17,21]. The relevance of the Ben-Lulu example is not that Jewish and Christian contexts are interchangeable, but that it illustrates the broader point that faith traditions do not engage sexuality education in a single fixed register. Religious pedagogy can be reinterpreted, and institutional boundaries around sexuality are historically negotiable.

The pastors’ emphasis on reputation, voice, mission, and audience also helps clarify why churches remain attractive sites for intervention. They possess forms of trust and regular access that many external programmes lack. Yet these same strengths may become limitations if youth are offered only a narrow or exclusionary sexual script. This is especially important when considering young people whose lives do not fit dominant church assumptions, including those who are already sexually active, those navigating coercion or violence, and queer youth who may find both school and church messaging inadequate or alienating [12].

The study therefore points less toward a simple endorsement or rejection of church involvement in AYSRH than toward the need for negotiated collaboration. Public health actors who engage churches must recognise the theological and moral worlds that shape how pastors interpret AYSRH. Conversely, churches seeking to contribute meaningfully to young people’s well-being may need to widen the scope of what counts as faithful care, beyond information-sharing framed only through abstinence and prohibition. More productive partnerships are likely to emerge where dialogue is honest about these differences rather than assuming that church infrastructure automatically translates into effective or inclusive AYSRH practice.

### 5.1. Limitations

This study has limitations that should be made explicit. First, the analysis is based exclusively on interviews with eleven pastors in one South African region. The article therefore reflects leadership perspectives rather than the lived experiences of adolescents, young adults, parents, or healthcare professionals. Second, the findings concern perceived assets and do not evaluate actual implementation, reach, acceptability, or impact on AYSRH outcomes. Third, although the sample was intentionally varied across church traditions and racial composition, the findings are not statistically generalisable. Their value lies instead in analytic and contextual transferability, meaning that readers may judge their relevance to comparable faith–health settings rather than treating them as universally representative. Fourth, because this article analyses one subset of a wider study, triangulation within the present paper is limited. These limitations mean that claims should remain close to the empirical scope of pastor interviews.

### 5.2. Implications for Research and Practice

Future research should examine how the assets identified here are interpreted by adolescents and young adults themselves, how parents and health professionals experience faith–public health partnerships, and whether specific church-based approaches produce supportive or exclusionary outcomes in practice. Comparative work across denominations, regions, and religious traditions would further help distinguish which dimensions of CHA are context-specific and which may travel more widely. For practice, the findings suggest that collaboration with churches should begin with explicit conversation about definitions of AYSRH, acceptable content, youth diversity, and referral pathways, rather than assuming consensus where it may not exist.

## 6. Conclusions

In conclusion, the pastors interviewed in the Vaal Triangle perceived churches to possess a rich repertoire of tangible and intangible assets relevant to AYSRH. These assets include infrastructure, networks, finances, social trust, moral authority, and embodied witness. However, the study also demonstrates that perceived assets are not automatically beneficial in themselves. Their public-health relevance depends on how they are interpreted, whose needs they prioritise, and whether they can engage young people’s realities without collapsing AYSRH into a single moral script. Churches may therefore be important partners in AYSRH, but their contribution should be understood as contingent, negotiated, and in need of empirical testing rather than assumed effectiveness.

## Figures and Tables

**Table 1:** Summary of the interviewed pastors.

Vaal Area	Pastor Pseudonym	Church Orientation	Congregational Racial Composition
Vanderbijlpark	P1	Open/accommodative (Anglican)	Mixed (predominantly White)
Sasolburg	P2	Pentecostal	Predominantly Black African
Vanderbijlpark	P3	Conservative	Predominantly Black African
Vanderbijlpark	P4	Conservative	Predominantly Black African
Vereeniging	P5	Conservative	Mixed (predominantly White)
Sasolburg	P6	Pentecostal	Predominantly Black African
Vanderbijlpark	P7	Conservative (Baptist)	Mixed (predominantly White)
Vanderbijlpark	P8	Pentecostal	Predominantly Black African
Vereeniging	P9	Pentecostal	Predominantly Black African
Sasolburg	P10	Pentecostal (African Pentecostal)	Predominantly Black African
Vanderbijlpark	P11	Open/accommodative (Anglican)	Mixed (predominantly White)

**Table 2:** Summary of tangible and intangible assets identified by Pastors.

Category	Asset	How Pastors Described It	Analytic Interpretation
Tangible	Church interaction spaces	Buildings, meeting venues, WhatsApp/Facebook, mentorship spaces	Churches were imagined as available platforms for encounter, education, refuge, and communication, although pastors often spoke hypothetically rather than describing established programmes.
Tangible	Community resources	Parents, health professionals, lay leaders, family structures	Churches were seen as reservoirs of relational and professional capital that could be activated for youth support.
Tangible	Normative teaching materials	Bible, study guides, manuals	Teaching resources were framed less as comprehensive health tools than as vehicles for moral direction and correction.
Tangible	Networks and partnerships	NGOs, health services, social workers	Partnerships were valued, especially where problems exceeded church capacity, but often as referral extensions rather than integrated co-design.
Tangible	Financial resources	Tithes, programme funding, camps, workshops	Financial capacity was uneven and was most often imagined as enabling events rather than sustained service delivery.
Intangible	Reputation	Trust, credibility, perceived truthfulness	Moral legitimacy increased the authority of church messages, but could also reinforce exclusivist claims about acceptable information.
Intangible	Voice on sexuality	Conscience of society, counselling, teaching	The pastoral voice combined support and moral regulation, reflecting both care and doctrinal discipline.
Intangible	Mission and vision	Duty to teach God’s way	AYSRH was incorporated into a theological obligation to guide conduct, especially toward abstinence.
Intangible	Ready audience	Regular church attendance and community reach	Pastors regarded congregations as accessible audiences for repeated messaging.
Intangible	Embodied messages	Testimonies and lived witness	Moral teaching was strengthened through example, repentance narratives, and visible church life.

## Data Availability

The qualitative interview data are not publicly available due to confidentiality and ethical restrictions but may be made available by the author on reasonable request.
